# Cardiovascular magnetic resonance feature tracking derived strain parameters in patients with acute myocarditis and preserved ejection fraction: a validation study

**DOI:** 10.1186/1532-429X-18-S1-W21

**Published:** 2016-01-27

**Authors:** Bettina Baessler, Frank Schaarschmidt, Laura Quellhorst, Anastasia Dick, Guido Michels, David Maintz, Alexander Bunck

**Affiliations:** 1 Department of Radiology, University Hospital of Cologne, Cologne, Germany; 2 Institute of Biostatistics, Faculty of Natural Sciences, Leibniz Universität Hannover, Hannover, Germany; 3 Department III of Internal medicine, Heart Center, University Hospital of Cologne, Cologne, Germany

## Background

The purpose of this study was to validate the diagnostic value of cardiovascular magnetic resonance (CMR) feature tracking (FT) derived strain parameters of both ventricles in patients with acute myocarditis (ACM) and preserved left ventricular ejection fraction (EF).

## Methods

CMR data of 18 patients with clinically suspected ACM and confirmation of diagnosis by CMR according to the Lake Louise criteria (LL criteria) were retrospectively analyzed. 30 healthy volunteers (HV) served as a control. A second cohort consisting of 51 patients with clinically diagnosed ACM and preserved EF served as a validation cohort. All patients and HV were examined on a clinical 1.5T scanner. Analysis of global longitudinal (long.), circumferential (circ.) and radial strain and strain rate (SR) of both ventricles was performed in one long-axis and three short-axis slices using a dedicated FT-software (TomTec Imaging Systems). Statistical analysis was conducted using independent t-test, one-way ANOVA with tukey-type comparisons, multinominal logistic regression analyses, classification trees, and ROC-analyses.

## Results

Patients with CMR-proven ACM and preserved EF (n = 18) showed a significantly improved basal RV circ. SR compared to HV (-0.76 ± 0.05 vs. -0.47 ± 0.07 s^-1^, p = 0.005) while LV strain parameters showed no significant differences between both groups. In multinominal logistic regression analyses, LV circ. strain and basal RV circ. SR proved to be the best independent predictors of ACM when LV-EF is preserved with an AUC of 0.82 in ROC-analysis. In classification trees, a cut-off of -29.0% for LV circ. strain and of - 0.43 s^-1^ for basal RV circ. SR resulted in 80% sensitivity and 80% specificity for classification between HV and CMR-proven ACM with preserved EF.

Applying these cut-offs on the validation cohort (n = 51) resulted in a moderate diagnostic sensitivity of 56% and a specificity of 71%. A re-estimation of logistic regression models in the validation cohort showed that a combination of LV long. strain and basal RV circ. SR was superior in predicting ACM compared to the model containing LV circ. strain. In classification trees, a cut-off of -20.4% for LV long. strain and of - 0.37 s^-1^ for basal RV circ. SR resulted in 77% sensitivity and 80% specificity for classification between HV and ACM with preserved EF.

**Figure 1:**
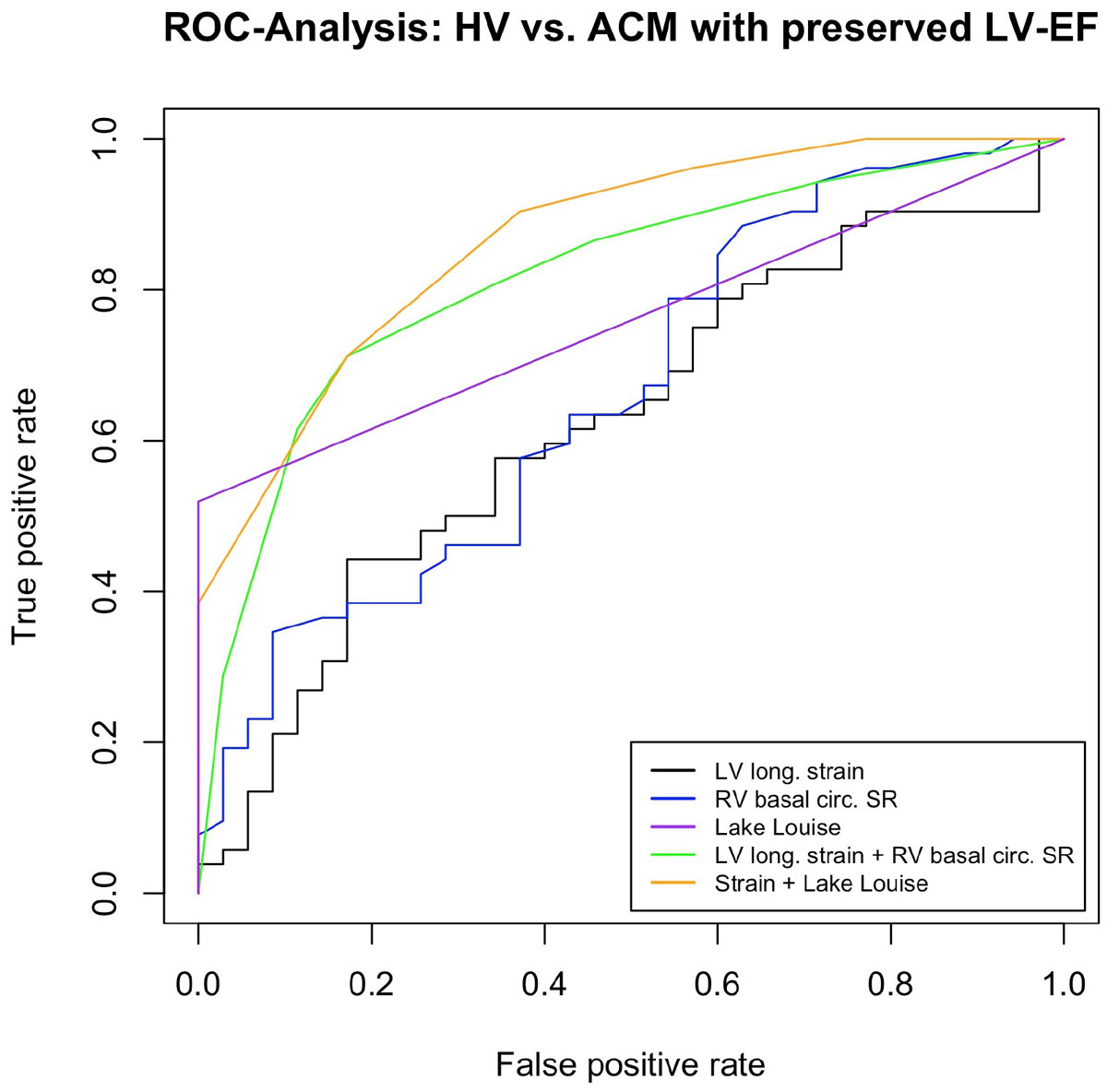
**ROC-analysis for differentiating healthy volunteers from ACM patients in the validation cohort**. Lake Louise Criteria (recorded as "present" or "not present"): Late Gadolinium Enhancement (LGE) + Visual edema on T2 black blood images + Early Gadolinium Enhancement

In ROC-analyses, the combination of LV long. strain and basal RV circ. SR showed superior diagnostic performance (AUC 0.82) when compared to LL criteria (AUC 0.76; Figure 1), and the combination of LV long. strain, basal RV circ. SR and Late Gadolinium Enhancement (LGE) further improved prediction of ACM with preserved EF with an AUC of 0.87.

## Conclusions

The proposed cut-off values for LV long. strain and basal RV circ. SR show an additional diagnostic value to the established Lake Louise criteria and may serve as novel diagnostic parameters in the setting of ACM.

